# Female Disorders

**Published:** 1886-08

**Authors:** Elias Wildman

**Affiliations:** Trenton, N. J.; Formerly Health Officer for Mt. Pindus Township, Michigan


### *FEMALE DISORDERS.*

A correspondent writes as follows respecting *Caulocorea*, advertised in our columns.

I meet constantly with many cases of female disorders in my daily practice, among which the most common are the dragging pains from the small of back and loins, the weakness and unsteadiness of limbs, a deranged condition of the digestive organs, loss of appetite, languor, flatulency, irregular action of bowels, headache, nervousness, loss of sleep, pevishness, and a constant mental dread as though something dreadful was about to occur. The patient being totally unfit for the cares and struggles of life, all these being caused by a disordered condition of the uterine functions. These troublesome symptoms have resisted all the usual modes of treatment. I have in a large experience, exhausted my supplies of remedies taken from all systems of medicine to promote a cure in severe cases, and often met with unsatisfactory results, till I tried Dr. Lowell's preparation of Caulocorea. This, I found to be the long needed remedy for Prolapsus (or Falling) Leuchorrhoae and Ulceration. I have used it daily for the past six months with great success; it may be taken by those who have delicate stomachs, without interfering with the digestive functions and in such cases I find it to improve the appetite. The patients gain in weight, and the skin regains its healthy color.

There are various so-called uterine tonics, but I am free to speak of Caulocorea as wholly reliable. It has been thorougly tested and is well worthy of recommendation.

Elias Wildman, M.D., D.D.S.
Trenton, N. J.

Formerly Health Officer for Mt. Pindus Township, Michigan.

